# The Predictive Validity of Machine Learning Models in the Classification and Treatment of Major Depressive Disorder: State of the Art and Future Directions

**DOI:** 10.3389/fpsyt.2020.00472

**Published:** 2020-05-25

**Authors:** Nick J. Ermers, Karin Hagoort, Floortje E. Scheepers

**Affiliations:** Department of Psychiatry, University Medical Center Utrecht, Utrecht, Netherlands

**Keywords:** electronic health records, major depressive disorder, machine learning, natural language processing, precision psychiatry, predictive modeling

## Abstract

Major depressive disorder imposes a substantial disease burden worldwide, ranking as the third leading contributor to global disability. In spite of its ubiquity, classifying and treating depression has proven troublesome. One argument put forward to explain this predicament is the heterogeneity of patients diagnosed with the disorder.

Recently, many areas of daily life have witnessed the surge of machine learning techniques, computational approaches to elucidate complex patterns in large datasets, which can be employed to make predictions and detect relevant clusters. Due to the multidimensionality at play in the pathogenesis of depression, it is suggested that machine learning could contribute to improving classification and treatment.

In this paper, we investigated literature focusing on the use of machine learning models on datasets with clinical variables of patients diagnosed with depression to predict treatment outcomes or find more homogeneous subgroups. Identified studies based on best practices in the field are evaluated. We found 16 studies predicting outcomes (such as remission) and identifying clusters in patients with depression.

The identified studies are mostly still in proof-of-concept phase, with small datasets, lack of external validation, and providing single performance metrics. Larger datasets, and models with similar variables present across these datasets, are needed to develop accurate and generalizable models. We hypothesize that harnessing natural language processing to obtain data ‘hidden' in clinical texts might prove useful in improving prediction models. Besides, researchers will need to focus on the conditions to feasibly implement these models to support psychiatrists and patients in their decision-making in practice. Only then we can enter the realm of precision psychiatry.

## Depression, Heterogeneous and Highly Subjective

Major depressive disorder, the most common of all diagnoses within psychiatry, imposes as substantial disease burden worldwide. With 300 million people worldwide suffering from depression, the disorder has indeed prevailed as one of the leading causes of health loss for nearly three decades and ranks as the third leading contributor to global disability (1–3). Despite its ubiquity, accurately diagnosing depression appears troublesome (3, 4). In a recent meta-analysis pooling studies conducted in 30 countries, the prevalence of depression as measured by self-report instruments was found to be considerably higher than based on clinical interviews (17.3% versus 8.5%) (3). Inaccurate assessment is considered an important barrier to effectively combatting depression, with primary care physicians, who are responsible for the majority of care in depression, identifying the disorder in only 50% of patients (4). Once the patient is diagnosed, the next challenge is selecting the appropriate treatment. In the STAR*D cohort, which includes more than 4,000 patients with major depressive disorder, treatment with citalopram, a current first-line antidepressant drug, led to remission in just 30% of patients (5). Moreover, approximately 30% of all patients did not remit after four consecutive treatments with antidepressants of different classes.

One avenue that is currently explored to improve treatment outcomes is through the development of novel therapies, such as deep brain stimulation and ketamine (6, 7). An alternative would be “treatment selection”—trying to find a specific treatment for each individual patient, among all available options, that is most likely to be effective (8). Such an approach recognizes that no single treatment is likely to be the best for everyone. It follows that the efficacies of drugs can be improved, as long as we accurately identify those patients that will respond well to that specific treatment. This strategy, coined “precision medicine,” has been applied in other areas of medicine and afforded major advances, particularly in cancer treatment (8).

One argument to explain the difficulties in correctly diagnosing and treating depression is the heterogeneity of the disorder (9). Throughout the history of medicine, similarities in clinical presentation have resulted in clustering “disease manifestations” into one disorder. With the advent of more advanced testing, heterogeneity is sometimes objectivated—a phenomenon than can be illustrated with the case of diabetes, with increasingly more subtypes being identified as our understanding of the pathophysiology advances. In contrast to many diseases with elucidated etiologies, the term ‘mental disorder' reminds us that we can solely observe “a syndromic constellation of symptoms that hang together empirically, often for unknown reasons” (10). Østergaard et al. demonstrated mathematically that the DSM-IV criteria for depression allowed for 1497 combinations of symptoms, and hence the authors state that “the current depressive syndrome is at least very, if not too heterogeneous” (11). This is not merely a theoretical conjecture; it is supported by research demonstrating that disease trajectories (and treatment responses) vary between different ‘depression subgroups’. For instance, one meta-analysis found that the magnitude of the benefit of antidepressants over placebo might be minimal or nonexistent in patients with mild depressive symptoms, whereas this benefit is substantial in patients with severe depression (12). Such findings underline the importance of stratifying patients into more appropriate, homogeneous subgroups, and adjusting their treatment regimes. Moreover, clinical trials, designed with strict inclusion and exclusion criteria, cannot account for the impact of individual nuances. Such nuances, however, can be crucial in a disorder that exists in so many different “shapes and sizes,” especially when the experience of symptoms as is highly subjective as is the case in mental illnesses.

## The Advent of Machine Learning

Recently, many areas of our daily life have been permeated by “machine learning,” among others in the shape of targeted marketing, speech recognition services and self-driving cars. Elucidating patterns and using that to improve predictions, has proven to be invaluable. This approach has not yet been widely adopted in the field of medicine, partly due to ethical and privacy concerns (13). However, machine learning models already demonstrated potential in various medical disciplines, such as cardiology and radiology (14, 15). The tremendous wealth of information stored in electronic health records, as well as the information that can be collected through new technologies like wearables, might offer immense potential in an area in which endless variability clouds our understanding of “disease mechanisms” (16).

Machine learning can be defined as “a computational strategy that automatically determines (“learns”) methods and parameters to reach an optimal solution” (17). Crucially, machine learning techniques take a data-driven approach: algorithms learn from examples without being explicitly programmed, which contrasts with more theory-driven approaches (10). The lack of hypotheses and “preselection” of variables could potentially allow for novel predictive associations that would otherwise go unnoticed.

Broadly speaking, machine learning techniques can be divided into two groups (18):

- Supervised learning, in which an algorithm is designed that takes candidate predictors to estimate an already defined, or “labeled”, outcome. For example, 12-month cancer survival could be predicted using features such as age, severity of symptoms, and blood parameters.
- Unsupervised learning, in which the aim is to separate unlabeled (“unclassified”) data into groups of related cases. Its goal is not to predict a predefined outcome, but, rather, to discover unknown “clusters” within the data. For example, it can be harnessed to define subgroups of patients that are similar in clinical presentation, and might thus have similarities in their etiology, prognosis and/or treatment efficacy. The identified clusters must then be interpreted to understand why (and how) the model designates their phenotypes as homogeneous.

One major risk in developing prediction models with machine learning techniques is known as “overfitting”, meaning that the model fits the data in the dataset used for training too precisely. It learns the “noise” of that specific dataset, in such a way that its predictions cannot be accurately replicated in other datasets (19). In order to prevent overfitting, and test whether a model can be of external value, models need to be designed according to rigorous standards. Gillian and Whelan enumerate best practices in their paper, which include (10):

- Studies involving machine learning approaches should apply internal (cross-)validation methods, to ensure that the model found in the training data can be extrapolated to unseen cases. This involves dividing the data set into training and test sets; the first is used to build the model, which is then tested in the latter.
- To ensure its generalizability the model also needs to be validated in an external dataset (external validation). In practice, this means that the model should only incorporate features that are widely available throughout different datasets.
- In order to have sufficiently-sized sets to train and test, large datasets are needed. When sample sizes are bigger, it becomes increasingly difficult to fit noise in the data, and more likely that the model actually captures the “signal” of the data.
- A range of performance metrics needs to be provided to quantify how well the model predicts the outcome. Area Under the Curve (AUC) is often named as the measure of choice in evaluating classification-learning algorithms, but cannot fully capture performance (particularly because it is not able to account for differences in base rates of response to treatment) (20).

## Machine Learning in Psychiatry

### The Promise of Machine Learning

The promise of achieving better prediction models and defining more meaningful “intermediate phenotypes” might be particularly valuable within psychiatry. In this field datasets of patients are usually of high dimensionality, combining different types of information, whether that be electronic health records, sociodemographic data, laboratory tests, genetics, or observations from imaging and real-world monitoring. Indeed, a recent meta-review summarized research that tried identifying predictors for (general) antidepressant response, and found 199 reviews (of many more original research papers) (21). Despite the multitude of identified predictors, the authors were not able to assess the direction and strength of these predictors due to methodological disparities and heterogeneity in effect sizes. They concluded that “despite so much work, very few (if any) predictors have entered clinical practice”.

Our traditional statistical methods (like unpenalized logistic regression and hypothesis testing) appear unable to elucidate complex patterns (such as interactions between predictors) and harness these to develop prediction models. More advanced machine learning techniques might offer a solution (22).

Models derived from machine learning techniques could, for instance, predict the chances of successful treatment with a certain antidepressant or the likelihood of treatment resistance for a specific patient.

Disease stratification and accurate predictions would allow for precision medicine, which contrasts with the “trial-and-error” approach that is still commonplace. Pioneering efforts are currently directed towards achieving this within psychiatry. Dwyer et al. reviewed numerous examples of machine learning applied to optimize diagnoses, prognoses and treatment outcome predictions within psychiatry, mainly focusing on imaging data (17). Once such models are optimized, they can be used to develop clinical decision support systems that can guide clinicians in making decisions tailored to every patient. Garg et al. found that practitioner performance (in different areas of medicine) was improved in approximately 60% of the reviewed 97 cases in which such clinical decision support systems were applied (23).

### Potential Caveats

Although not in the scope of this work, it is imperative that we briefly consider some of the pitfalls of applying machine learning in medical practice. Cabitza et al. distinguish the following (24):

- When confronted with computer-aided detection, the diagnostic sensitivity of clinicians is in some cases reduced. Assuming the model does its work, could make the clinician as a “second assessor” interpret his own observations with less prudence.
- The “demise of context”: Because of a focus on what can be translated into data, information that cannot be fitted within machine learning models might be pushed to the background. Especially in psychiatry this could be problematic, since symptoms are experienced in a subjective manner and the narratives giving context to symptoms are of major importance.
- The “black-box problem”: When models become increasingly more complex and multidimensional, the relationship between predictors and outcomes might become incomprehensible and untraceable. The model might in a particular instance recommend a certain treatment, without clinicians being able to understand how the computer arrived at its conclusion. This “opacity” might hinder the uptake of models in daily practice.
- Observer variability, inherent to medical diagnoses, is often not incorporated into machine learning models. The observations fed into the model are treated as truth, rather than what they really are: approximations of reality, inevitably containing errors.

### Assessing Machine Learning Models

This perspective article aims to narratively review research in which datasets of patients with depression were analyzed with machine learning techniques, to create prediction models or define clusters. Our overarching goal is to assess whether the identified studies met best practices in the field of machine learning, and whether machine learning models are likely to be implemented into clinical practice in the forthcoming years. Broadly, in this review the following types of studies are considered:

- Studies predicting outcomes (such as remission or resistance after treatment) in patients diagnosed with depression, based on supervised learning methods.
- Studies predicting outcomes based on “interpreting” electronic health records. An approach to valorize this information is through natural language processing, an automated method to process written records. This technique can be harnessed to extract (or encode) clinical concepts from texts based on a set of rules (25).
- Studies identifying relevant clusters within an aggregate of patients diagnosed with depression, based on unsupervised learning methods.

Predictors for depression can be categorized into “clinical” variables, that can be readily obtained during a clinical interview or examination, and “biological” variables, that require additional efforts (such as taking blood samples or imaging). For reasons of practicality, we have decided to solely focus on models that consider clinical variables - and thus excluded studies that consider biomarkers or neuroimaging in their model. In a clinical setting, it is not (yet) feasible to collect that type of data for every patient diagnosed with depression—among others for financial reasons—and thus their relevance in a decision-support system is not as evident as information that can be obtained during standard psychiatric examination (10).

## State of the Art: What Has Been Done?

We conducted a bibliographic search on the PubMed and EMBASE databases for articles containing the keywords “depression” and “machine learning” and their synonyms. Articles were included if they investigated the use of machine learning approaches in predicting treatment outcomes or find more homogeneous clusters (in an adult patient population). Searching the databases resulted in 2,277 unique records, 72 remained after title and abstract screening, and 16 studies were included in our analysis after full-text screening (26–41). The results of these studies (and the techniques that were used) are reported in the supplementary tables, below we narratively highlight the key findings.

### Predicting Outcomes From Clinical Variables

We found 10 studies that investigate clinical variables to predict outcomes (**Supplementary Table 1A**). Sample sizes of these studies varied considerably; between 116 and 2,555 patients were included. Not all studies included the number of variables, but the ones that did, ranged from 9 to 48 variables. Next to clinical and (socio)demographic features, some researchers also looked at other variables, such as early symptom change (27). The identified studies used very different performance metrics to gauge accuracy; indeed, the performance metrics provided could differ within a study (e.g. between training and test sample). For the sake of comparison, we will use the AUC to approximate the accuracy of the models, when available.

Most studies assessed response, often defined as reaching a certain cut-off score on one of two commonly used scales to assess depression severity (i.e. the Hamilton Rating Scale for Depression and the Quick Inventory of Depressive Symptomatology). This approach gives dichotomous outcomes (“response” versus “no response”). Serretti et al. found that using these dichotomous outcomes resulted in higher accuracy than stratifying response in multiple classes (26). This intuitively makes sense, because accurately predicting to what extent one will respond must be harder than simply predicting whether one will respond at all. No other research is known to have attempted this “response stratification” approach. Neither did any try to define response as a certain change on the scales, so that rather than reaching the cut-off value, the amount of improvement would be considered—which appears more meaningful than reaching an artificial boundary on these scales.

All included studies did perform internal validation, mostly 10-fold cross-validation. However, only two out of the ten studies fulfilled the “best practice” requirement of validating their model in an external dataset (30, 35).

The study by Chekroud et al., predicting response to treatment with citalopram, was the first to provide a wide range of performance metrics (with an AUC of 0.700), and also validated their model both internally and externally (the latter with data from a clinical trial) (30). Interestingly, there was modest evidence that their prediction model could be generalized to remission after treatment with escitalopram. However, the model failed to predict response to other antidepressants (i.e. combination therapy of venlafaxine and mirtazapine).

In addition to remission, Iniesta et al. also predicted treatment-resistance, reaching an AUC of 0.67 (31). Predicting resistance could prove useful as those patients can be “fast-tracked” to alternative treatment options. Another study attempted to predict chronicity of depression, with various outcomes such as “number of years with depression” (32). Participants were reinterviewed 10 to 12 years after their initial interview, and the models for the various outcomes reached AUCs ranging from 0.63 to 0.76.

The model that included most variables (48 in total) to predict remission and resistance, appeared to perform well, but could not be compared to other studies, as it used accuracy (between 0.737 and 0.850) rather than AUC (33). In another study by the same authors, it was investigated what the effect of a reduction of the included features was on the accuracy of the model (34). The authors constructed two models, including 47 and 15 variables respectively; accuracy decreased from 75.0% to 71.0% in their training sample.

Interestingly, four studies assessed the added value of harnessing “advanced” machine learning techniques in classifying patients, by comparing the performance of their model to the accuracy of a logistic regression model (26, 29, 32, 41). The machine learning models outperformed the logistic regression in three of these studies (26, 32, 41).

### Harnessing Natural Language Processing to Predict Outcomes

Two studies similarly utilized clinical data, but obtained this (partially) through natural language processing (**Supplementary Table 1B**). Huang et al. used baseline clinical features in combination with unstructured clinical texts, to predict treatment response, for antidepressants and psychotherapy (36). In contrast to the studies in the previous section, it was not elaborated on which variables were included in the model. A much larger sample size was used, with 5,651 patients included; the model reached AUCs of 0.661 and 0.749 for predicting response to antidepressant treatment and psychotherapy respectively. Another study, also with a large sample size (n = 4,687), predicted psychiatric readmission from electronic health records (37). By integrating data obtained through natural language processing into the model (rather than solely using baseline clinical features), the AUC improved from 0.618 to 0.784.

### Distinguishing Depression Clusters Using Clinical Variables

Four studies used unsupervised learning algorithms to investigate whether clusters can be found within the “unitary construct” of patients with depression (**Supplementary Table 1C**); in all, three clusters were identified.

One study used hierarchical clustering to identify “clinical profiles” (i.e. combinations of clinical characteristics) and assessed remission and response rates across these profiles (28). Response rates across profiles ranged from 31% to 63% (47% in the overall population) and remission rates ranged from 12% to 55% (28% overall). They concluded that these profiles were more useful than individual factors for predicting outcomes of antidepressant treatment. In addition, they also found that socioeconomic indicators were the most important and “had greater overall predictive power” than depressive symptoms and comorbidities.

Two studies used World Mental Health diagnostic surveys among patients with depression, to define clusters. Van Loo et al. found three clusters (high, intermediate, and low risk) based on index episode symptoms, with the high-risk cluster (consisting of 30% of all patients) accounting for 53%–71% of high persistence/severity (38). Elaborating on the findings from that study, Wardenaar et al. found that including comorbidities in their analysis, resulted in the high-risk cluster (32,4%) accounting for 56.6–72.9% of high outcomes (39).

Chekroud et al. identified three clusters of symptoms based on correlations within the QIDS and HAMD scales: sleep (symptoms of insomnia), core emotional (symptoms relating to mood, energy, interest and guilt), and atypical (suicidality, hypersomnia, psychomotor slowing and agitation) (40). They then reevaluated the efficacy of antidepressants in seven clinical trials, to investigate whether the observed clusters have different response trajectories. Antidepressants were found to be most effective for the cluster with (predominantly) core emotional symptoms, less so for sleep symptoms and least for atypical symptoms.

## Discussion

### State of the Art

The models we discussed in this paper used clinical variables to predict outcomes of depression treatment or find meaningful clusters within heterogeneous patient samples. From reviewing the literature, it can be asserted that most studies are still in the proof-of-concept phase. The models are created and validated in small samples. Besides, in all but two studies external validation was not performed, thus risking that the model might fit well to the dataset used for training and testing, but cannot be extrapolated to other patients. Additionally, the majority of studies just provided one performance measure, whereas best practice requires multiple metrics.

Nonetheless, applications might be possible in the near future. The studies using purely clinical features reached AUCs between 0.63 and 0.78, comparable to the models using natural language processing techniques. Kessler and colleagues compared the AUC they found (0.76 for their best performing model) with other risk models used within medicine: 0.74 for a widely used prediction score for coronary heart disease (Framingham Risk Score) and typically below 0.70 for models to predict the course of breast cancer. They consequently stated their model might be of relevance in clinical practice (32).

Moreover, the clusters identified as “high risk” were indeed shown to have higher chances on worse outcomes, and response trajectories for antidepressants were found to differ among clusters. These findings suggest that defining subgroups might improve care by anticipating disease trajectories and differentiating in treatment choices.

### Future Directions

In accordance with best practices, future studies should aim to test their models in large, independent samples and provide various performance measurements. In reality, this might turn out difficult, as the variables obtained in clinical practice often differ widely between treatment centers. In addition, future models should attempt to make more meaningful predictions by using multiple and categorical (or continuous) outcomes, rather than the dichotomous classes that the studies discussed here have used.

Crucial to choosing the right variables and outcome measures, as well as to eventually achieving the implementation of the models in practice, is the involvement of medical staff (42). Which variables are easily attainable and could serve as potential features? What would encourage clinicians to make use of the model? Also privacy issues need to be considered when collaborating with other centers to increase the amount of data or perform external validation. One possibility to collaboratively work on models while avoiding sharing of patient data, is through “federated learning” (43). In this approach, the model is available for use in different centers through for example a cloud service and data is not integrated.

Importantly, models should be trained and tested on patient data despite the variety of clinical practice. Machine learning techniques allow for the increased heterogeneity in non-trial data, which is necessary to make the model applicable to patients outside of the strict inclusion and exclusion criteria of trials.

The studies discussed in this paper, aimed at response prediction to one antidepressant, instead of comparing different interventions. Ideally, attempts should be directed at finding so-called “moderators,” that predict different responses for multiple treatments (44). Two studies have previously attempted to generate individualized treatment recommendations through the use of a “Personal Advantage Index” (45, 46). These studies produced predictions of post-treatment severity for each patient in each of the two interventions (e.g. antidepressant medications versus cognitive behavioral therapy). The comparison of these predictions yields an index that shows which treatment will produce the best outcome.

Notably, we found that sample sizes of studies utilizing natural language processing were considerably larger, which serves as an argument to augment models with data obtained through this technique. In their review, Ford and colleagues enumerate some more benefits of harnessing information from clinical texts: it is more engaging, allows for the expression of feelings, and is a “better reminder for the clinician of the human encounter” (47). Structured information may be too limiting, and leaves no space for nuances. Free text becomes particularly relevant when findings do not exactly match “codeable” symptoms or diagnoses, or when contexts matters. Moreover, text could specifically be of value since clinical notes are widely available throughout different treatment centers.

Importantly, before machine learning models can be implemented within psychiatry, a consensus needs to be reached, stipulating when a model can be considered “clinically relevant.” According to American Psychiatric Association a biomarker needs to have 80% accuracy, before it has “clinical utility” (48). Gillan & Whelan argue, however, that this threshold eventually comes down to a cost/benefit trade-off: How much do we win and lose when we apply this model (10)? Most studies did not compare the performance of their model to the “performance” of clinicians. This might be due to the nature of psychiatry, in which diagnosing is difficult and accuracy varies between studies, raters and over time. Just one study verified whether their tool improved predictions beyond the performance of clinicians—be it in a small, online survey (30). A total of 23 clinicians completed their predictions about treatment response in a sample of 26 patients, and their performance was compared to the machine learning model. The latter did markedly better, in terms of accuracy (46,3% versus 69,2%). Before any clinical decision support system can be implemented, treatment allocation based on algorithm-guided assignment (possibly in conjunction with a psychiatrist) needs to be compared more thoroughly to physician-guided treatment. However, whether reaching accurate predictions regarding antidepressant efficacy is a feasible goal to strive for at all should also be questioned. Not everything a patient goes through in his/her path to recovery, can be measured or recorded; and thus be plugged into our models. This might give rise to unexplained variation, causing our models to underperform, or fail in the long run. In a disorder that is interwoven with all life's complexity, biological and social, we could not have expected differently.

Two more obstacles exist in the implementation of treatment prediction models (49):

- First, the burden that collecting the information required for the risk calculation might pose on clinicians and patients. The number of features exceeded twenty in many models. Collecting patient information and inserting this into a calculation tool would evidently be time-consuming. Also, some of the models take data from different questionnaires—meaning that all need to be filled out before the model can make predictions. Again, models harnessing natural language processing to analyze clinical texts might prove valuable. These models use data from clinical summaries that are written during routine examination, and thus do not require drastic changes in the diagnostic process.
- Secondly, another challenge will be to turn the very complex statistical models into easy-to-use (and understand) applications, as the accessibility of clinical decision support system is a major determinant in the uptake of such tools. A promising development is that some researchers have actually published online tools that allow clinicians to access the models from their workplace.

This article sought to present an overview of the application of machine learning techniques to improve the classification and treatment of depression. We conclude that many hurdles need to be overcome before prediction models will have their place within standard clinical practice. This will not just entail fine-tuning the models and increasing their accuracy, by using larger datasets and externally validating their results. Researchers will also need to tackle questions on how such models can be implemented, for instance by reducing the features that have to be acquired for every patient. Only then we can move away from the “paradigm of average efficacies” and enter the realm of precision psychiatry, where individual predictions based on patient characteristics are reality.

## Author Contributions

NE was responsible for conception and design of the article, and drafted the manuscript. All authors contributed to manuscript revision, read and approved the submitted version.

## Conflict of Interest

The authors declare that the research was conducted in the absence of any commercial or financial relationships that could be construed as a potential conflict of interest.
