# Supplementary material for: The Predictive Validity of Machine Learning Models in the Classification and Treatment of Major Depressive Disorder: State of the Art and Future Directions
Source: Front Psychiatry. 2020 May 25;11:472. doi: 10.3389/fpsyt.2020.00472 (PMC7261928; doi:10.3389/fpsyt.2020.00472)
Supplement: Supplementary file 1 [file DataSheet_1.pdf]

Table 1a: Overview of studies, in order of publication. Studies included in this table employed supervised learning techniques to create prediction models from clinical variables.

First author, year	Data utilized	Sample size and diagnosis; if applicable, intervention received	Machine learning model employed	Definition of outcome measure(s), time frame	Provided performance metrics	Cross-validation performed?	Remarks
**Serretti, 2007**	- Clinical and demographic features<br>- 15 variables incorporated into model	- 116 inpatients diagnosed with depression<br>- Treated with fluvoxamine	- Artificial neural network	- Response after 6 weeks *(defined as a decrease to ≤8 on HAM-D-21 score with no delusion)*	Training sample<br>- 90% correctly classified<br>Test sample<br>- 62% correctly classified<br>- AUC: 0.769<br>[Only metrics of best performing model displayed]	- Split-sample validation	- Outperformed model using logistic regression<br>- Model with dichotomous outcome measurement performed better than using multiple outcome classes
**Kuk, 2010**	- Baseline features<br>- Early symptom change<br>[Number of variables incorporated into model not provided]	- 2,280 outpatients diagnosed with depression, from STAR*D cohort<br>- Treated with citalopram	- Recursive subsetting	- Response after 6 weeks *(defined as ≥ 50% reduction in QIDS-SR score)*	Training sample<br>- NPV: 0.80<br>- Overall PPV not mentioned	- 10-fold cross-validation in training sample	- Using baseline features alone did not result in model with clinically useful predictions
**Perlis, 2013**	- Clinical and sociodemographic variables (self-reported)<br>- 15 variables incorporated into model	- 2,555 patients diagnosed with depression, from STAR*D cohort<br>- Treatment with citalopram, and, if unsuccessful, second level treatment with another antidepressant	Various:<br>(a) Naïve Bayes<br>(b) Support Vector Machine<br>(c) Random Forest	- Remission after treatment with antidepressants (at level 1 or 2) *(defined as a score of ≤ 5 on QIDS-SR)*	Training sample, AUC:<br>(a) 0.716<br>(b) 0 .706<br>(c) 0.697<br>Test sample, AUC:<br>(a) 0.698<br>(b) 0.693<br>(c) 0.706	- 10-fold cross-validation in training sample<br>- Internal validation with test sample (n=523)	- Machine learning models were compared with logistic regression model, the latter performed better<br>- Simple calculator was constructed from the best performing model (i.e. logistic regression)

Chekroud, 2016	- Patient-reportable variables<br>- Total of 25 variables (from 164) incorporated into model	- 1,949 patients diagnosed with depression, from STAR*D cohort<br>- Treated with citalopram (level 1)	- Elastic net regularization for feature selection<br>- Gradient boosting machine	- Remission after 12 weeks *(defined as a score of ≤ 5 on QIDS-SR)*	Training sample<br>- AUC: 0.700<br>- Accuracy: 64.6%<br>- Sensitivity: 62.8%<br>- Specificity: 66.2%<br>External dataset<br>- Accuracy: 59.6%<br>- Sensitivity: 49.4%<br>- Specificity: 70.8%<br>[AUC not provided]	- 10-fold cross-validation in training sample<br>**- Externally validated in escitalopram group of COMED trial (n = 151)**	- Validation in other treatment arms of COMED trial not considered here
Iniesta, 2016	- Demographic and clinical variables<br>- 41 variables incorporated into model for remission<br>[Not provided for resistance]	- 793 patients diagnosed with depression, from GENDEP cohort<br>- Treated with nortriptyline or escitalopram	- Elastic net regularized regression	- Remission *(defined as HAM-D-17 score of ≤ 7)*<br>- Resistance *(defined as lack of response after two antidepressants)*	Training sample<br>*Remission:*<br>- AUC = 0.72<br>- Sensitivity = 0.66<br>- Specificity = 0.66<br>*Resistance:*<br>- AUC = 0.67<br>[Other outcome metrics not provided]	- 10-fold cross-validation in training sample	- Models with other outcomes not considered here
Kessler, 2016	- Baseline lay-administered interviews, from WMH surveys<br>- Re-interview after 10-12 years<br>- 9-13 variables incorporated into models for different outcomes	- 1,056 patients diagnosed with depression	- Ensemble regression trees,<br>- Penalized regression	Numerous:<br>- High persistence *(episode lasting 2+ weeks)*<br>- High chronicity *(an episode lasting most days throughout the year)*<br>- Hospitalization<br>- Disability *(50% limitation in performing paid work)*<br>- Attempted suicide	Training sample<br>AUC for all outcome measures ranged from 0.63 to 0.76	- 10-fold cross-validation in training sample	- Consistently outperformed logistic regression, despite using less predictors

Kautzky, 2017a	- Clinical features<br>- 48 variables incorporated into model	- 480 patients diagnosed with depression, from GSRD cohort	- Random Forest	- Treatment resistance *(defined as HAM-D-17 score of ≥ 17 after 2 antidepressants)*<br>- Remission *(defined as HAM-D-17 score of < 8)*	<u>Training sample</u><br>- Accuracy resistance: 0.737<br>- Accuracy remission: 0.850<br><u>Test sample</u><br>- Accuracy resistance: 0.737<br>- Accuracy remission: 0.850	- Internal validation with test sample (n = 80)	- Highest accuracy was achieved when all variables were combined
Kautzky, 2017b	- Clinical and sociodemographic features<br>- 47 variables incorporated into model<br>**- A simplified model with 15 features was also designed**	- 522 patients diagnosed with depression, from GSRD cohort	- Random Forest	- Treatment resistance *(defined as failure to reach response after 2 antidepressants [≤ 22 on MARDS])*	<u>Training sample</u><br>- Accuracy: 75.0%<br>- Sensitivity: 82.2%<br>- Specificity: 62.5%<br>**- Accuracy reduced to 71.0% with 15 features**<br><u>Test sample</u><br>- Accuracy of 70.6% for simplified model<br>[Other outcomes not provided]	- 10-fold cross-validation in training sample<br>- Internal validation with test sample for simplified model	- Highest accuracy was achieved when all variables were combined
Nie, 2018	- Clinical features from enrollment<br>- Level 1 treatment response (up to week 2)<br>[Number of variables incorporated into model not provided]	- 2,454 patients diagnosed with depression, from STAR*D cohort	- Random Forest, GBDT, XGBoost, L2 penalized logistic regression, elastic net	- Remission *(defined as a score of ≤ 5 on QIDS-SR after level 1 or 2 treatment)*	<u>Test sample</u><br>- AUC: 0.70-0.78<br><u>External dataset</u><br>- AUC: 0.72-0.77	- 10-fold cross-validation in training sample<br>- Testing sample (20%)<br>**- Externally validated in RIS-INT-93 cohort *(remission defined as a score of ≤ 7 on HAM-D-17)***	

**Hatton, 2019**	Baseline demographic and psychometric data [Number of variables incorporated into model not provided]	284 patients (older adults with 'subthreshold depression'), from CASPER trial	- XGBoost	- Persistence of depressive symptoms at 12 months *(defined as a score of ≥ 10 on PHQ-9)*.	Accuracy: 74%<br>AUC: 0.72<br>Sensitivity: 0.78<br>Specificity: 0.56<br>PPV: 0.89<br>NPV: 0.35	- 60:40 split for training/test set, with 1000 iterations	Outperformed model using logistic regression

Table 1b: Overview of studies, in order of publication. Studies included in this table employed natural language processing and subsequently supervised learning techniques to create prediction models from clinical texts.

First author, year	Data utilized	Sample size and diagnosis; if applicable, intervention received	NLP & machine learning model employed	Definition of outcome measure(s)	Provided performance metrics	Cross-validation performed?	Remarks
**Huang, 2014**	- Age, gender, baseline PHQ-9 score<br>- Unstructured clinical texts	- 5,651 patients diagnosed with depression, from GHRI cohort<br><br>(a) 2,472 patients taking antidepressant medication<br>(b) 2,401 patients receiving psychotherapy	- NLP: NCBO annotator<br>- LASSO	Response to treatment, *(defined as a 5-point decrease in PHQ-9 score)*	(a) AUC = 0.661<br>- using 133 features<br><br>(b) AUC = 0.749<br>- using 193 features on average	- Trained on 80% of sample, tested on 20%	- Most predictive feature = baseline PHQ-9 score<br>- No statistically significant moderators for differential response<br>- Topics incorporated in model not elaborated on
**Rumshisky, 2016**	- Baseline clinical features<br>- Narrative electronic health record discharge summaries	- 4,687 inpatients diagnosed with depression	- NLP : Latent Dirichlet Allocation (LDA) model<br>- Support Vector Machine	- Psychiatric readmission within 30 days	Testing sample<br>- AUC: 0.784	- 3-fold cross-validation in training sample<br>- Internal validation with 30% of patients	- AUC for baseline clinical features was lower, i.e. 0.618

Table 1c: Overview of studies, in order of publication. Studies included in this table employed unsupervised learning techniques to identify clusters with high similarity within the whole sample.

First author, year	Data utilized	Sample size and diagnosis	Machine learning model employed	Clusters identified; evaluation of the clusters
**Jain, 2013**	Demographic and clinical variables	- 2876 patients diagnosed with depressing, from STAR*D cohort	- Recursive quality receiver operating characteristics<br>- Hierarchical clustering	Outcome: response to escitalopram *(defined as ≥ 50% improvement on QIDS-SR)* and remission *(defined as HAM-D-17 ≤ 7 at study exit, or last QIDS-SR ≤ 5)*<br><br>- Identified 'clinical profiles' based on clinical features (among others income, interest in activities and insomnia). Response rates ranged from 31 to 63% (47% overall) and remission rates ranged from 12 to 55% (28% overall) across profiles<br>- The clinical profile associated with highest probability of remission (55%): less depressed mood, at least minimal interest in activities and income of $40.000 or more
**Van Loo, 2014**	World Mental Health (WMH) fully-structured diagnostic surveys	- 8,261 respondents with lifetime depression	- k-means cluster analysis<br>[- Ensemble recursive partitioning, LASSO generalized linear model]	- Clustering based on symptoms of index episode (early onset, suicidality, anxiety, dysphoria)<br>- Outcome parameters: years in episode, years in chronic episode, hospitalization, disability<br><br>- 3 clusters identified: high, intermediate, low risk<br>- AUCs for different outcomes range from 0.61-0.72<br>- High-risk cluster (30.0% of cases) accounted for 53-71% of high persistence/severity
**Wardenaar, 2014**	World Mental Health (WMH) fully-structured diagnostic surveys	- 8,261 respondents with lifetime depression	- k-means cluster analysis<br>[- Regression tree analysis, lasso, ridge, elastic net penalized regression]	- Clusters based on comorbidities<br>- Outcome parameters: years in episode, years in chronic episode, hospitalization, disability<br><br>- 3 clusters identified: high, intermediate risk, low risk<br>- AUCs for different outcomes range from 0.62-0.73<br>- High risk cluster (32.4% of cases) accounted for 56.6-72.9% of high outcomes

Chekroud, 2017	- Clinical and sociodemographic features<br>- 25 variables incorporated into prediction models (different for each cluster)	- 4,039 patients diagnosed with depression, from STAR*D cohort	- Hierarchical clustering<br>[- Elastic net, gradient boosting machine]	Three clusters identified, within both the QIDS-SR and HAM-D-17 scales:<br>- core emotional symptoms<br>- sleep (insomnia) symptoms<br>- atypical symptoms<br><br>Clusters were then used to:<br>- Assess differential response trajectories of the symptoms within a cluster<br>- Construct outcome predictions for each cluster<br>[Results not included here]<br><br>- For clustering: COMED trial (n = 640)<br>- For prediction models: 7 randomized controlled trials<br><br>- Web-based clinical decision tool was constructed based on findings of the study

Abbreviations used: **AUC** = area under receiver-operating characteristic curve; **CASPER** = Collaborative Care and Active Surveillance for Screen-Positive Elders with subthreshold depression; **COMED** = Combining Medications to Enhance Depression Outcomes; **GBDT** = gradient boosting decision tree; **GENDEP** = Genome-Based Therapeutic Drugs for Depression; **GSRD** = Group for the Study of Resistant Depression; **HAM-D-17** = 17-item Hamilton Rating Scale for Depression; **HAM-D-21** = 21-item Hamilton Rating Scale for Depression; **LASSO** = least absolute shrinkage and selection operator; **MARDS** = Montgomery-Åsberg Depression Rating Scale; **NCBO** = National Center for Biomedical Ontology; **NLP** = Natural Language Processing; **NPV** = negative predictive value; **PHQ-9** = patient health questionnaire-9; **PPV** = positive predictive value; **QIDS-C** = 16-item Quick Inventory of Depressive Symptomatology, clinician-rated; **QIDS-SR** = 16-item Quick Inventory of Depressive Symptomatology, self-reported; **RIS-INT-93** = Janssen clinical study (NCT00044681); **STAR*D** = Sequenced Treatment Alternatives to Relieve Depression; **VAS** = visual analogue scale; **XGBOOST** = extreme gradient boosting.
